# Treatment of *in vitro*-Matured Bovine Oocytes With Tauroursodeoxycholic Acid Modulates the Oxidative Stress Signaling Pathway

**DOI:** 10.3389/fcell.2021.623852

**Published:** 2021-02-19

**Authors:** Elisa Mariano Pioltine, Camila Bortoliero Costa, Laís Barbosa Latorraca, Fernanda Fagali Franchi, Priscila Helena dos Santos, Gisele Zoccal Mingoti, Fabíola Freitas de Paula-Lopes, Marcelo Fábio Gouveia Nogueira

**Affiliations:** ^1^Multi-user Laboratory of Phytomedicines Pharmacology, and Biotechnology (PhitoPharmaTec), Institute of Biosciences, Department of Pharmacology, São Paulo State University, Botucatu, Brazil; ^2^Department of Biological Sciences, Federal University of São Paulo, Diadema, Brazil; ^3^School of Veterinary Medicine, Department of Production and Animal Health, São Paulo State University, Araçatuba, Brazil; ^4^Laboratory of Embryonic Micromanipulation, School of Sciences and Languages, Department of Biological Sciences, São Paulo State University, Assis, Brazil

**Keywords:** oocyte, *in vitro* maturation, TUDCA, endoplasmic reticulum stress, oxidative stress, cattle

## Abstract

In several species, oocyte and embryo competence are improved by the addition of endoplasmic reticulum (ER) stress inhibitors to *in vitro* maturation (IVM) medium and/or *in vitro* culture (IVC) medium. This study aimed to evaluate the effects of three concentrations of tauroursodeoxycholic acid (TUDCA; 50, 200, and 1,000 μM), a chemical chaperone for relieving ER stress, during IVM of bovine cumulus–oocyte complexes (COCs) for 24 h. Treated oocytes were analyzed for nuclear maturation, reactive oxygen species (ROS) production, mitochondrial activity, and abundance of target transcripts. In addition, the number of pronuclei in oocytes was evaluated after 18–20 h of insemination, and the rates of blastocyst and hatched blastocyst formation were evaluated after 7 and 8/9 days of culture, respectively. We further evaluated the transcript abundance of embryonic quality markers. Our findings showed that supplementation of IVM medium with 200 μM of TUDCA decreased ROS production and increased abundance of transcripts related to antioxidant activity in oocytes (*CAT*, *GPX1*, and *HMOX1*) and embryos (*GPX1* and *PRDX3*). Interestingly, high concentration of TUDCA (1,000 μM) was toxic to oocytes, reducing the nuclear maturation rate, decreasing mitochondrial activity, and increasing the abundance of ER stress (*HSPA5*) and cellular apoptosis (*CASP3* and *CD40*) related transcripts. The results of this study suggest that treatment with 200 μM of TUDCA is associated with a greater resistance to oxidative stress and indirectly with ER stress relief in bovine oocytes.

## Introduction

*In vitro* maturation (IVM) is one of the main restrictive steps in the optimization of *in vitro* production (IVP). During IVM, oocytes acquire the intrinsic capacity for gradual development until activation of the embryonic genome after fertilization (Ferreira et al., 2009; Gilchrist, 2011).

However, previous studies have indicated that *in vitro* conditions in which oocytes are exposed to a variety of cellular stresses contribute to the greater incidence of loss of competence in *in vitro* developed embryo compared with that in embryos derived *in vivo* (Latham, 2016; Del Collado et al., 2017). Response to exogenous stress is a vital part of cellular physiology and it is increasingly becoming apparent that one of the main mechanisms involved in initiating the cellular response to a variety of exogenous stressors is associated with the endoplasmic reticulum (ER; Guzel et al., 2017). ER is an important organelle responsible for protein folding, transport and synthesis, trafficking, metabolism of lipids, and cellular Ca^2+^ storage (Groenendyk and Michalak, 2005; Hetz, 2012). *In vitro*, however, the ER microenvironment can be disturbed due to Ca^2+^ depletion, hypoxia, and N-terminal glycosylation dysfunction, causing ER stress (Hetz, 2012). ER stress is triggered when misfolded or unfolded proteins accumulate in the lumen of the ER. As a pro-survival response, unfolded protein response (UPR) alleviates the accumulation of unfolded proteins and restores ER function (Schröder and Kaufman, 2005). However, when ER stress exceeds its threshold, cellular damages such as apoptosis, degeneration, and carcinogenesis ensue (Olzmann et al., 2013; Yoon et al., 2014).

It is worth mentioning that oxidative stress and reactive oxygen species (ROS) generation are key to ER stress and not just consequences of its induction (Bhandary et al., 2012; Yoon et al., 2014). In addition, the exacerbated increase in ROS levels resulting from ER stress can cause the amplification of mitochondrial ROS and, consequently, the activation of pro-apoptotic signaling pathways (Sutton-McDowall et al., 2016; Fan and Simmen, 2019).

To relieve ER stress, ER stress inhibitors are added to the culture media. Tauroursodeoxycholic acid (TUDCA), a bile acid that acts as a potent chemical chaperone (Xie et al., 2002; Cortez and Sim, 2014), has been used to alleviate ER stress during *in vitro* oocyte maturation and/or embryo development (Song et al., 2011; Kim et al., 2012; Zhang et al., 2012; Yoon et al., 2014; Zhao et al., 2015; Mochizuki et al., 2018). Although the exact chaperoning mechanism of TUDCA is still unclear, it has been shown to prevent UPR malfunction and ameliorate ER stress in various cell types (Xie et al., 2002; Miller et al., 2007; Lee et al., 2010; Seyhun et al., 2011). However, little is known about the mechanism of TUDCA activity and its effects on *in vitro*-matured bovine oocytes.

We hypothesized that IVM of bovine oocytes supplemented with TUDCA could relieve the ER stress caused by the *in vitro* environment. Thus, we proposed a model to improve oocyte maturation and, consequently, fertilization and *in vitro* embryonic development.

Therefore, the aim of this study was to evaluate the effect of different TUDCA concentrations on *in vitro* oocyte maturation and the quality, polyspermy, and *in vitro* blastocyst development in cattle.

## Materials and Methods

All chemicals used in this study were purchased from Sigma–Aldrich (St. Louis, MO, United States) unless otherwise indicated.

### Experimental Design

**FIGURE 1:**
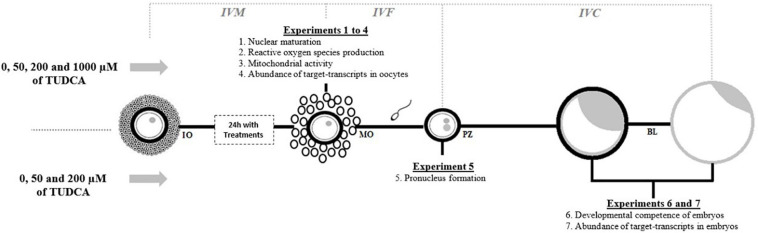
Illustrative experimental design. IVM, *in vitro* maturation; IVF, *in vitro* fertilization; IVC, *in vitro* culture; IO, immature oocyte; MO, mature oocyte; PZ, presumptive zygote; BL, blastocyst and hatched blastocyst.

Tauroursodeoxycholic acid (Selleckchem, Houston, TX, United States) was dissolved in sterile distilled water to prepare a 100 mM stock solution that was stored at −80°C. This stock solution was freshly diluted with IVM media to obtain 50 μM (T50), 200 μM (T200), and 1,000 μM (T1000) solutions of TUDCA (Kim et al., 2012; Zhang et al., 2012; Mochizuki et al., 2018; Figure 1). In all experiments, we included the control group (without TUDCA). The first series of experiments aimed to evaluate the influences of control, T50, T200, and T1000 TUDCA on the following oocyte quality parameters during IVM of bovine oocytes: nuclear maturation (experiment 1), ROS production (experiment 2), mitochondrial activity (experiment 3), and the abundance of target transcripts in oocytes (experiment 4). Other experiments aimed to evaluate the influence of supplementation with control, T50, and T200 TUDCA during IVM on fertilization and embryonic development, particularly on pronucleus formation in presumptive zygotes (experiment 5), developmental competence of embryos (experiment 6), and transcript abundance of embryonic quality markers (experiment 7).

### *In vitro* Maturation

Bovine ovaries, from *Bos taurus indicus* and its crossbreeds, were obtained from a commercial abattoir located in Assis (São Paulo state, Brazil; latitude: 22°36’38.78’ S and longitude: 50°26′53.54′′ W). These ovaries were transported to the laboratory in sterile saline (0.9% NaCl) at 37°C for 30 min at maximum. *Cumulus*–oocyte complexes (COCs) were collected by the aspiration of follicles with 3–8 mm diameter (Botigelli et al., 2018). After sedimentation, COCs were recovered and selected using a stereomicroscope. Only COCs with a homogeneous cytoplasm and a compact multilayer of *cumulus* cells were used (grades 1 and 2; Seneda et al., 2001). COCs were washed and transferred to 500 μL drops of maturation medium (10 μL/COCs) in a four-well plate consisting of TCM199 with Earle salts and supplemented with 0.1 IU/mL rhFSH (Gonal-f, Merck Serono, Rockland, MA, United States), 0.22 mg/mL sodium pyruvate, 75 μg/mL amikacin, 4 mg/mL bovine serum albumin (BSA), and increasing concentrations of TUDCA according to the experimental design previously described (section “Experimental Design”). All experiments had a control group. COCs were incubated at 38.5°C in humidified air with 5% CO_2_ for 24 h.

### *In vitro* Fertilization and Culture

In experiments where *in vitro* fertilization (IVF) and *in vitro* culture (IVC) were performed, groups of 25 COCs were transferred to 90 μL drops of Tyrode albumin lactate pyruvate (TALP) supplemented with fatty-acid-free BSA (6 mg/mL), pyruvate (0.22 mg/mL), amikacin (75 μg/mL), heparin (30 μg/mL), and PHE (20 μM penicillamine, 10 μM hypotaurine, and 1 μM epinephrine). Oocytes were inseminated with frozen-thawed semen from a single sample of a Nelore breed bull. Spermatozoa were selected using the Select SPERM (Botupharma Animal Biotechnology, Botucatu, São Paulo, Brazil) method, and the concentration was adjusted to 1 × 10^6^ spermatozoa/mL. Oocytes and spermatozoa were co-incubated under the same conditions as during IVM, and the day of insemination was designated as Day 0. At 18–20 h post-insemination, presumptive zygotes were denuded from *cumulus* cells and transferred to 500 μL drops of SOF medium (synthetic oviduct fluid; 10 μL/zygotes) in a four-well plate, supplemented with pyruvate (0.22 μg/mL), amikacin (75 μg/mL), 2.5% v/v fetal calf serum, and BSA (5 mg/mL). Subsequently, the oocytes and spermatozoa were cultivated under physiological oxygen tension (5%) and high humidity in small sealed plastic bags with a gas mixture containing 5% O_2_, 5% CO_2_, and 90% N_2_ (based on Vajta et al., 1997) in an incubator at 38.5°C. In all experiments, the culture was maintained for 9 days after insemination until the hatching stage of the embryos. Blastocyst and hatched blastocyst formation rates were assessed as the proportions of observed structures on days 7 and 8/9, respectively, based on the number of COCs used in IVM.

### Nuclear Staining

Nuclear staining was used in two studies. COCs (Experiment 1) and presumptive zygotes (Experiment 5) were collected after 24 h of IVM and 18–20 h of IVF, respectively, and then vortexed in wash medium for 2 min. Denuded oocytes and presumptive zygotes were fixed in 4% (v/v) paraformaldehyde for 30 min in a humidifier chamber at room temperature (RT), incubated with 5 μg/μL Hoechst 33342 for 30 min at RT, and transferred to Poly-L-lysine-coated slides mounted with a coverslip. The oocytes and presumptive zygotes were analyzed using an epifluorescence inverted microscope (Eclipse Ti-E, Nikon, Japan) with A4 filter (emission 420 nm and excitation 330–385 nm). Nuclear maturation was graded into two categories: immature oocytes (germinal vesicle stage and metaphase I without first polar body) and mature oocytes [metaphase II (MII) with primary polar body; Ghaffari Novin et al., 2015]. Pronucleus formation was graded into three categories: (1) unfertilized oocytes with a single pronucleus in the ooplasm, (2) fertilized oocytes with two pronuclei in the ooplasm, and (3) polyspermic oocytes with more than two pronuclei in the ooplasm (Zhao et al., 2018).

### Evaluation of Reactive Oxygen Species and Mitochondrial Activity

For Experiments 2 and 3, the same staining protocol was performed and the details of each one are provided in the text below. COCs were collected at 0 and 24 h of IVM and vortexed in wash medium for 2 min. The denuded oocytes were separately incubated in 5 μM Cell Rox Green (Life Technologies, Foster City, CA, United States) or 0.5 μM Mito Track Red CMX ROS (Invitrogen, Ltd.) for 30 min in a humidifier chamber at 38.5°C or RT, depending on the purpose of the study. Then, the oocytes were fixed in 4% (v/v) paraformaldehyde for 15 min in a humidifier chamber at RT and transferred to Poly-L-lysine-coated slides mounted with a coverslip. In the staining, fixation, and slide mounting processes, oocytes were washed three times in phosphate-buffered saline (PBS) containing 1 mg/mL polyvinylpyrrolidone (PVP). Oocytes were analyzed using an epifluorescence microscope (Eclipse Ti-E, Nikon) equipped with an L5 filter (emission 519 nm and excitation 495 nm) for ROS detection and with N21 filter (emission 615 nm and excitation 587 nm) for mitochondrial evaluation. In both experiments, a digital camera attached to the microscope was used to acquire images and the fluorescent pixel intensity value of the total area of each oocyte was measured using a freehand tool to delimitate the cytoplasm of each oocyte (ImageJ software^1^). Background fluorescence was subtracted from each image before fluorescence measurement and quantification (Rodrigues et al., 2016; Ispada et al., 2018).

### Relative Quantitation of Target-Transcripts: Reverse Transcription Quantitative Polymerase Chain Reaction (RT-qPCR)

#### RNA Isolation and Reverse Transcription

Total RNA from oocytes and blastocysts was extracted using the PicoPure RNA Isolation kit (Life Technologies, Foster City, CA, United States) following the manufacturer’s protocol. Extracted RNA was stored at −80°C until further analysis by qPCR. RNA concentration was quantified using a spectrophotometer (Nanodrop, Thermo Fisher Scientific, Waltham, MA, United States).

For each sample, we used a pool of 20 oocytes and a pool of three blastocysts for reverse transcription. cDNA synthesis was performed using a High Capacity Reverse Transcription kit (Applied Biosystems, Foster City, CA, United States), following the manufacturer’s instructions. All samples were treated with DNase according to the manufacturer’s instructions before reverse transcription.

#### Pre-amplification and qPCR

Gene expression analyses of bovine oocytes and blastocysts were performed independently using Applied Biosystems^TM^ TaqMan^®^ Assays specific for *B. taurus* and based on Fontes et al. (2020). A total of 86 target genes were analyzed (Supplementary Table S1 describing all the genes and their signaling pathways). Prior to qPCR thermal cycling, each sample was subjected to sequence-specific preamplification process as follows: 1.25 μL assay mix (TaqMan^®^ Assay was pooled to a final concentration of 0.2× for each of the 96 assays), 2.5 μL TaqMan PreAmp Master Mix (Applied Biosystems, #4391128), and 1.25 μL cDNA (5 ng/μL). The reactions were activated at 95°C for 10 min, followed by denaturation at 95°C for 15 s, annealing, and amplification at 60°C for 4 min for 14 cycles. These preamplified products were diluted fivefold (oocyte and embryos) prior to RT-qPCR analysis.

Assays and preamplified samples were transferred to an integrated fluidic circuit plate. For gene expression analysis, the sample solution preparation consisted of 2.25 μL cDNA (preamplified products), 2.5 μL of TaqMan Universal PCR Master Mix (2×, Applied Biosystems), and 0.25 μL of 20× GE Sample Loading Reagent (Fluidigm, South San Francisco, CA, United States); the assay solution included 2.5 μL 20× TaqMan Gene Expression Assay (Applied Biosystems) and 2.5 μL of 2× Assay Loading Reagent (Fluidigm). The 96.96 Dynamic Array^TM^ Integrated Fluidic Circuits (Fluidigm) chip was used for data collection. After priming, the chip was loaded with 5 μL each of the assay solution and each sample solution and loaded into an automated controller that prepares the nanoliter-scale reactions.

The qPCR thermal cycling was performed in the Biomark HD System (Fluidigm) using the protocol TaqMan GE 96 × 96 Standard, which involved one stage of Thermal Mix (50°C for 2 min, 70°C for 20 min, and 25°C for 10 min) followed by a hot start stage (50°C for 2 min and 95°C for 10 min), 40 cycles of denaturation (95°C for 15 s), primer annealing, and extension (both at 60°C for 60 s).

### Statistical Analysis

The fluorescence intensity data for ROS detection and mitochondrial activity were compared using the non-parametric Kruskal–Wallis test and Dunn’s *post hoc* test. Data on nuclear maturation rate, sperm penetration rate, and the rates of formation of blastocysts and hatched blastocysts were arcsines transformed and subjected to analysis of variance, and the means were compared using Tukey’s *post hoc* test. The normality of data was assessed using the Shapiro–Wilk and Bartlett tests. The results are presented as the mean ± standard error of the mean (SEM) or median, and first and third interquartile intervals based on data normality. Quantitative PCR data were assessed using the ΔC_q_ values relative to the geometric mean of the best reference genes among the 96-gene set, i.e., *HPRT1*, *PPIA*, and *HPRT1* (Experiment 4) and *GAPDH*, *HPRT1*, and *PPIA* (Experiment 7). Fold-changes were calculated using the 2^–ΔΔCq^ method. All analyses were performed using JMP software (SAS Institute, Cary, NC, United States). Moderate statistical significance was determined based on 0.01 < *P*-value ≤ 0.06, while strong significance was considered when *P*-value ≤ 0.01.

## Results

### The Effect of TUDCA on Oocyte Nuclear Maturation During IVM

**FIGURE 2:**
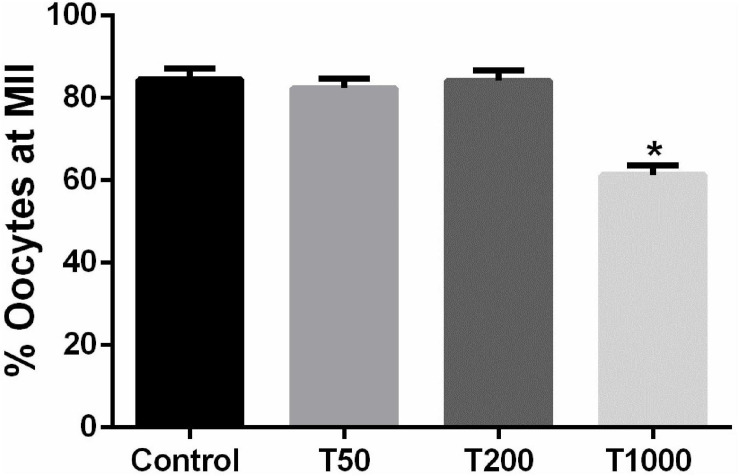
Effect of TUDCA concentrations on the proportion of oocytes that reached the MII stage during IVM. Results are represented by means ± SEM of five replicates using 87–110 COCs/treatment. The bar marked with asterisk differs significantly (*P* ≤ 0.06). Control, 0 μM TUDCA; T50, 50 μM TUDCA; T200, 200 μM TUDCA; T1000, 1,000 μM TUDCA.

After 24 h of IVM, the proportion of oocytes that reached MII was similar among the Control, T50, and T200 groups (Figure 2). However, the T1000 group exhibited decrease (*P* = 0.002) in the proportion of oocytes in MII compared to those in the other groups, with or without TUDCA treatment.

### The Effect of TUDCA on Oocyte ROS Production During IVM

**FIGURE 3:**
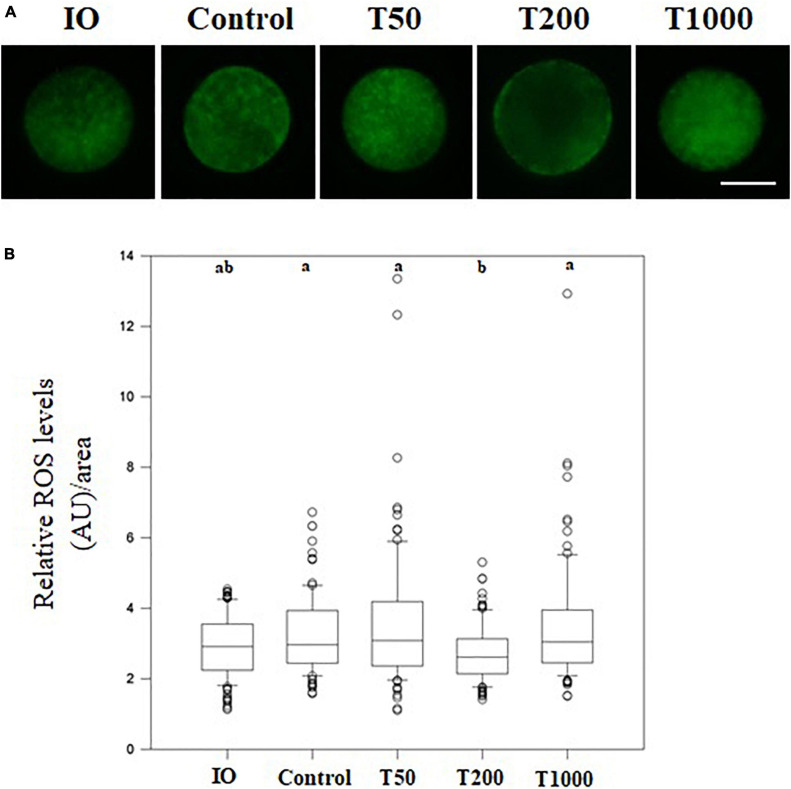
Intracellular ROS levels in oocytes matured *in vitro* in the presence of increasing TUDCA concentrations. **(A)** Representative photomicrographs of bovine oocytes stained with Cell Rox Green for ROS semi-quantitative quantification. Bar = 50 μm. **(B)** Results are presented as the median and first and third interquartile interval of five replicates using 87–95 COCs/treatment. Different letters in each box represent significant differences (*P* ≤ 0.06). IO, immature oocyte; Control, 0 μM TUDCA; T50, 50 μM TUDCA; T200, 200 μM TUDCA; T1000, 1,000 μM TUDCA.

After 24 h of IVM, the T200 group exhibited decreased ROS production (*P* = 0.001) compared to the other groups, with or without TUDCA treatment. There was no difference in ROS levels among the control, T50, and T1000 groups. Furthermore, immature oocytes were not significantly different from mature oocytes (Figure 3).

### The Effect of TUDCA on Oocyte Mitochondrial Activity During IVM

**FIGURE 4:**
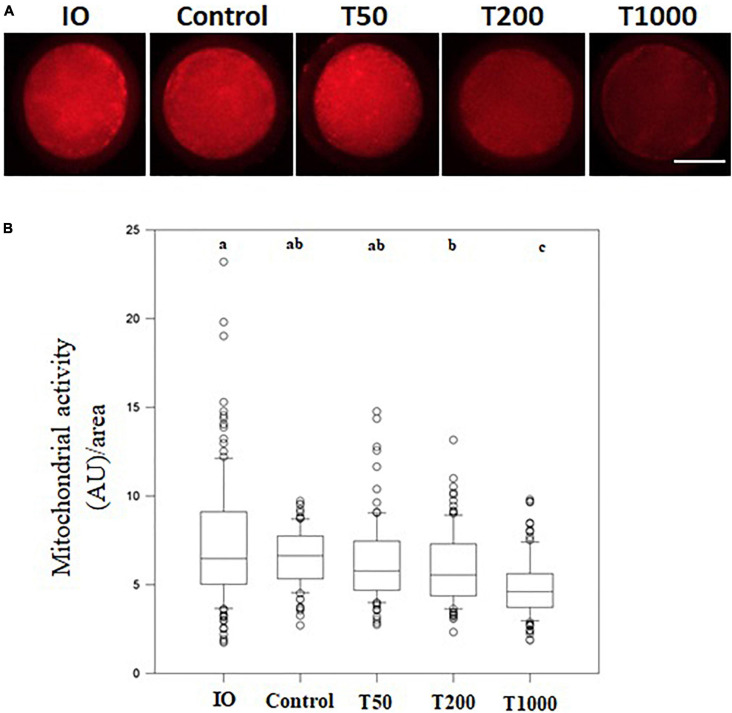
Mitochondrial activity in oocytes matured *in vitro* in the presence of increasing TUDCA concentrations. **(A)** Representative photomicrographs of bovine oocytes stained with Mito Track Red CMX ROS for mitochondrial activity semi-quantitative quantification. Bar = 50 μm. **(B)** Results are presented as the median and first and third interquartile interval of five replicates using 87–105 COCs/treatment. Different letters in each box represent significant differences (*P* ≤ 0.06). IO, immature oocyte; Control, 0 μM TUDCA; T50, 50 μM TUDCA; T200, 200 μM TUDCA; T1000, 1,000 μM TUDCA.

After 24 h of IVM, there was no difference in mitochondrial activity among the Control, T50, and T200 groups. However, the T1000 group showed decreased (*P* = 0.001) mitochondrial activity compared to the other groups, with or without TUDCA treatment. In addition, when matured oocytes were compared to immature oocytes (no IVM, i.e., oocytes just after being retrieved from ovarian follicles), treatments with 200 and 1,000 μM of TUDCA significantly reduced mitochondrial activity in the matured oocyte (*P* = 0.001; Figure 4).

### The Effect of TUDCA on the Abundance of Target-Transcripts in Oocytes During IVM

**FIGURE 5:**
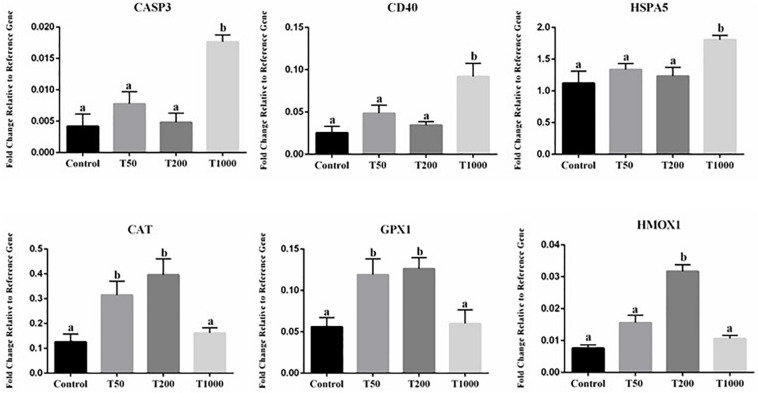
Effect of TUDCA concentrations on differential gene expression in oocyte during IVM. Data represent the fold change in level of expression relative to the reference gene. Results are represented by least-squares means ± SEM of five replicates using 100 COCs/treatment. Different letters in each bar represent significant differences (*P* ≤ 0.06). Control, 0 μM TUDCA; T50, 50 μM TUDCA; T200, 200 μM TUDCA; T1000, 1,000 μM TUDCA.

Transcript abundance of six genes in oocytes was significantly affected after TUDCA treatment (Figure 5). All the others target transcripts analyzed (Supplementary Table S1) did not differ in a statistically significant way. Compared with the Control group, transcript abundance was upregulated in the T50, T200, and T1000 groups, according to each gene evaluated. Among the differentially expressed genes, there were transcripts related to ER stress (*HSPA5*) and oxidative stress, in which the T1000 group was upregulated; response to cellular stress (*CAT*, *GPX1*, and *HMOX1*) was upregulated in the group treated with 200 μm of TUDCA; and apoptosis (*CASP3* and *CD40*), which the T1000 group was upregulated (Figure 5).

### The Effect of TUDCA Supplementation During IVM on Pronucleus Formation in Presumptive Zygotes and Developmental Competence of Embryos

A total of 237 oocytes were evaluated (79, 74, and 84 oocytes, respectively, for the Control, T50, and T200 groups). The rates of pronucleus formation in the oocytes with one, two, or more pronuclei were not statistically different among the Control, T50, and T200 groups (*P* = 0.79; Table 1). There was no significant difference in the rates of formation of blastocysts (*P* = 0.14; Table 2) and hatched blastocysts on days 7 and 8/9, respectively, after fertilization (*P* = 0.96; Table 2).

**TABLE 1:** Pronuclei formation in Control, T50, and T200 groups.

	Control	T50	T200	*P*
**Total number of oocytes**	79	74	84	
**Unfertilized oocytes rate (%; mean ± SEM)^a^**	19.43 ± 1.42	20.36 ± 4.10	17.07 ± 2.97	**0.79**
**Fertilized oocytes rate (%; mean ± SEM)^a^**	74.68 ± 2.12	75.61 ± 3.34	77.19 ± 1.04	**0.76**
**Polyspermic oocytes rate (%; mean ± SEM)^a^**	5.89 ± 2.49	4.03 ± 2.80	5.74 ± 2.44	**0.86**

*^*a*^Total pronuclei/presumptive zygote. Number of replicates: 5. Control, 0 μM of TUDCA; T50, 50 μM of TUDCA; T200, 200 μM of TUDCA. Bold value indicates non significant (NS).*

**TABLE 2:** Embryo production in Control, T50, and T200 groups.

	Control	T50	T200	*P*
**Total number of oocytes**	223	224	218	
**Blastocyst formation rate (%; mean ± SEM)^a^**	37.79 ± 0.85	43.10 ± 2.00	42.04 ± 2.32	**0.14**
**Hatched blastocyst formation rate (%; mean ± SEM)^b^**	37.49 ± 6.01	38.07 ± 6.61	35.60 ± 3.62	**0.96**

*^*a*^Total blastocysts/total oocyte—from D7. ^*b*^Total hatched blastocysts/total blastocysts—from D8 and D9. Number of replicates: 5. Control, 0 μM TUDCA; T50, 50 μM TUDCA; T200, 200 μM TUDCA. Bold value indicates non significant (NS).*

### The Effect of TUDCA Supplementation During IVM on Transcript Abundance of Embryonic Quality Markers

**FIGURE 6:**
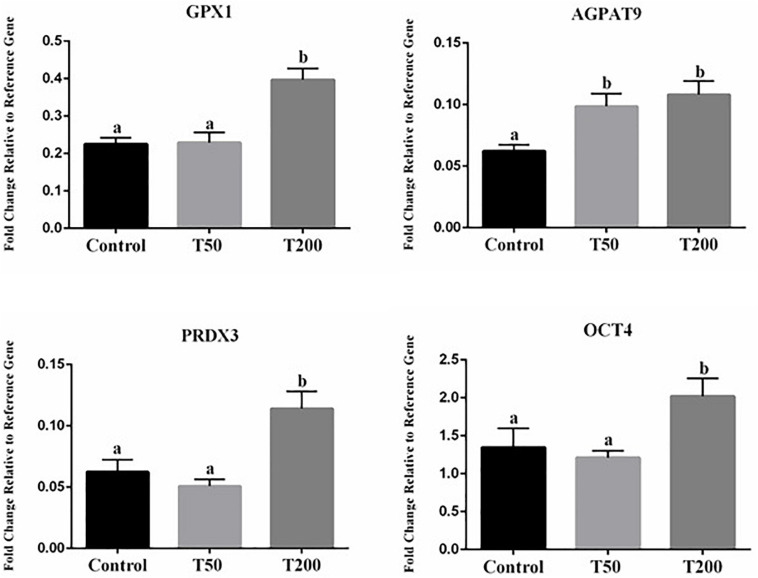
Effect of TUDCA concentrations on differential gene expression in embryo during IVM. Data represent the fold change in level of expression relative to reference gene. Results are represented as least-squares means ± SEM of five replicates using 218–224 COCs/treatment. Different letters in each bar represent significant differences (*P* ≤ 0.06). Control, 0 μM TUDCA; T50, 50 μM TUDCA; T200, 200 μM TUDCA.

Transcript abundances of four genes were significantly affected in blastocysts after TUDCA treatment during IVM (Figure 6). All the others target transcripts analyzed (Supplementary Table S1) did not differ in a statistically significant way. Compared with the control group, transcript abundances of embryonic quality markers were upregulated in the T50 and T200 groups. Among the differentially expressed genes, there were transcripts related to oxidative stress and response to cellular stress (*GPX1* and *PRDX3*), that were upregulated in the T200 group; metabolism (*AGPAT9*) being upregulated in T50 and T200 groups; and pluripotency and cell differentiation (*OCT4*) with higher relative abundance in T200 (Figure 6).

## Discussion

Overproduction and accumulation of ROS overloads the antioxidant defense mechanism, resulting in cellular damage called oxidative stress (Menezo et al., 2016). There is an intimate relation between ER stress and oxidative stress (Stadtman et al., 2002; Plaisance et al., 2016). Based on this relationship, the present study demonstrated that supplementing the IVM medium with TUDCA may relieve ER stress by decreasing ROS production in the oocyte and increasing the abundance of transcripts related to antioxidant activity in bovine oocytes and embryos developed *in vitro*.

Although ER is a key organelle involved in protein and lipid synthesis (Groenendyk and Michalak, 2005; Hetz, 2012), it is sensitive to disturbances in cellular homeostasis and can be triggered by different types of stresses (Latham, 2016), such as oxidative stress (Malhotra and Kaufman, 2007; Yoon et al., 2014). Such imbalance in cellular homeostasis leads to the accumulation of malformed proteins, activating ER stress and impairing cell function (Olzmann et al., 2013; Ghemrawi et al., 2018). In several species, it has been observed that ER stress is present during *in vitro* embryo production (Song et al., 2011; Kim et al., 2012; Zhang et al., 2012; Yoon et al., 2014; Zhao et al., 2015; Mochizuki et al., 2018), reducing oocyte competence and impairing embryonic development. In this study, we investigated the effects of supplementing IVM with different TUDCA concentrations during bovine oocyte maturation under *in vitro* environmental stress. Based on previous reports (Song et al., 2011; Kim et al., 2012; Zhang et al., 2012; Yoon et al., 2014; Zhao et al., 2015; Mochizuki et al., 2018), we propose a method to relieve ER stress in oocytes through treatment with TUDCA, reinforcing that ER stress is present in *in vitro-*matured oocytes.

Previous studies have demonstrated that TUDCA has potential therapeutic benefits on various models of many diseases, and its therapeutic effects are mainly attributed to its ability for relieving ER stress (Lee et al., 2010; Kusaczuk, 2019). In addition, it has been discovered that TUDCA reduces oxidative stress, suppresses apoptosis, and decreases inflammation in many *in vitro* models (Rodrigues et al., 1998; Schoemaker et al., 2004; Yoon et al., 2014; Rosa et al., 2017; Kim et al., 2019), thus demonstrating its antioxidant activity. Since oxidative stress can cause reduction–oxidation (redox) imbalance and trigger ER stress (Malhotra and Kaufman, 2007; Yoon et al., 2014), thereby increasing the excessive production of ROS and exacerbating oxidative stress (Malhotra and Kaufman, 2007; Yoon et al., 2014), we evaluated the function of TUDCA in modulating ROS levels in oocytes. During IVM, ROS levels were significantly decreased in COCs treated with 200 μM TUDCA compared to those in the control. Recently, a study has shown that 100 μM TUDCA supplementation reduces ROS production in bovine oocytes, which is in line with the findings of the present study (Khatun et al., 2020). In pigs, the use of 50 μM TUDCA during IVM reduced the ROS levels in oocytes (Zhang et al., 2012). Collectively, the data suggest that the mechanism of action of TUDCA in oocytes during IVM may be highly influenced by the species/subspecies, the tested concentration, and the *in vitro* conditions that the oocytes are subjected to, thus explaining the different results of previous studies and our present study. However, regardless of species and cell type, most studies have reported potential reduction in ROS levels due to TUDCA treatment (Rodrigues et al., 1998; Yoon et al., 2014; Yun et al., 2018), corroborating our findings.

Although the mechanistic details of TUDCA activity in the ER stress pathway have not been fully elucidated, previous studies have ascribed the beneficial role of this drug to suppression of UPR pathways (Kusaczuk, 2019), thus improving protein folding and reducing the expression of ER stress markers in several cell types (Xie et al., 2002; Seyhun et al., 2011; Kusaczuk, 2019). Based on this information, the abundances of transcripts related to oocyte and embryonic competence after oocyte maturation with TUDCA treatment were evaluated. In oocytes, a significant increase in the abundances of *CAT* and *GPX1* mRNAs was observed in the T50 and T200 groups, while *HMOX1* mRNAs was increased in the T200 group when compared with those in the control. The relative abundances of *CAT*, *GPX1*, and *HMOX1* mRNAs are related to the antioxidant defense mechanism, which is important for maintaining cellular homeostasis in cases of high ROS levels (Combelles et al., 2009; Di Emidio et al., 2014). Balance between ROS production and antioxidant cell defense mechanisms is essential during IVM; otherwise, oxidative stress could cause cell damage and apoptosis (Remião et al., 2016; Gad et al., 2018; Nie et al., 2020). Furthermore, in the embryo, a significant increase in the abundances of *GPX1*, *PRDX3*, and *OCT4* mRNAs was observed in the T200 group compared with those in the control. Moreover, the T200 group exhibited upregulation of genes related to the antioxidant mechanism, in addition to that of genes involved in cellular pluripotency. Similar to *GPX1*, *PRDX3* plays an important role in oocyte maturation and embryonic development (Gad et al., 2018; Pereira et al., 2018; Camargo et al., 2019). OCT4, encoded by the *POU5F1* gene, is a key component of the pluripotency regulatory network (Wu and Schöler, 2014). OCT4 is postulated to play a critical role in defining totipotency and inducing pluripotency during embryonic development (Wu and Schöler, 2014; Daigneault et al., 2018), and is essential for the retention of pluripotency in the inner cell mass and epiblast. We also found that the AGPAT9 isoform, a key regulator of lipid accumulation in adipocytes (Bunel et al., 2015), was upregulated in the T50 and T200 groups, suggesting that it can influence the highest lipid droplet content in the embryo.

In parallel with our findings, other experimental studies have reported beneficial effects of TUDCA as an antioxidant (Moreira et al., 2017; Rosa et al., 2017; Daruich et al., 2019). The function of TUDCA in relieving oxidative stress has been demonstrated through the high expression of NRF2, DJ-1, and the antioxidant enzymes heme oxygenase-1 (HO-1) and glutathione peroxidase (GPx) in the human neuroblastoma cell line SH-SY5Y (Moreira et al., 2017), and by the reduction of high ROS levels in various cell types (Rodrigues et al., 1998; Yoon et al., 2014; Yun et al., 2018).

Surprisingly, in contrast with the effects observed so far, 1,000 μM TUDCA was apparently toxic to bovine oocytes during IVM. Remarkable increases in the expression of the apoptosis markers CASP3 and CD40 and the ER stress marker HSPA5 were observed in oocytes of the T1000 group when compared to those of the control. HSPA5 is an important molecular chaperone that activates UPR pathways in response to ER stress (Hetz, 2012; Olzmann et al., 2013). High expression of HSPA5 is associated with low oocyte competence (Wu et al., 2012; Park et al., 2018). In addition, increased *HSPA5* mRNA abundance is associated with cell apoptosis in cases of severe ER stress, which could promote the expression of pro-apoptotic genes (Xie et al., 2002; Szegezdi et al., 2003, 2006), such as Caspase-3 (Xie et al., 2002). Similar to *CASP3*, positive regulation of *CD40* in oocytes is associated with low competence and death of the oocyte (Tatone et al., 2006; Pang et al., 2018).

Thus, we hypothesize that high concentrations of TUDCA can activate the classic mitochondrial apoptosis pathway. Previous reports have shown that severe ER stress can interfere with mitochondrial activity, contributing to the decoupling of oxidative phosphorylation, reduction of mitochondrial membrane potential, matrix swelling, and subsequent release of various apoptotic factors, including cytochrome C and effector caspases that lead to cellular apoptosis (Berridge, 2002). These effects on mitochondria demonstrate the importance of balancing the flow of Ca^2+^ between these organelles (Bentov et al., 2011; Wu et al., 2012), because imbalance in one of them, such as ER stress, can severely affect the other. In mice, it was observed that mitochondrial dysfunction in oocytes can be mitigated by treatment of ER stress, such as through the use of Salubrinal (an ER stress inhibitor) in IVM (Wu et al., 2012). Contrary to this report but complementing the gene expression results, the T1000 group showed decreased mitochondrial activity compared to the control group, suggesting that high concentration of TUDCA may activate ER stress and mitochondrial apoptotic pathways.

Reported studies in available literature indicate that increased mitochondrial activity is necessary for nuclear maturation and completion of meiosis II (Picton et al., 1998; Tarazona et al., 2006). In the T1000 group, the nuclear maturation rate decreased in comparison to that in the control group, corroborating that high concentration of TUDCA is harmful for *in vitro-*matured bovine oocytes. The detrimental effect of 1,000 μM TUDCA on bovine oocytes under IVM has not been previously reported.

Although treatments with 50 and 200 μM TUDCA were beneficial for IVM, nuclear maturation and mitochondrial activity did not differ from those in the control. Possibly, during ER stress, TUDCA (at optimal concentrations) has a preventive effect in the early stages of apoptosis that do not involve mitochondria, as suggested by previous studies that did not observe beneficial effects of TUDCA on mitochondrial activity (Xie et al., 2002).

As already mentioned, balancing calcium oscillations is of great importance for maintaining cellular communication (Whitaker, 2006; Wu et al., 2012). In mammals, calcium oscillation is a feature of fertilization and plays a central role in activating development. The calcium required for these oscillations is mainly derived from the ER, wherein it accumulates in clumps in the microvillar subcortex during oocyte maturation (Whitaker, 2006). ER migration to the cortex plays an important role in making the ER competent for generating calcium oscillations during oocyte maturation (Ducibella and Fissore, 2008). However, in stressful situations, ER distribution is altered, and calcium oscillations are impaired, making sperm penetration difficult (Ajduk et al., 2008). In our study, the addition of TUDCA to the maturation medium was not able to modulate oocyte fertilization, as demonstrated by the pronuclei number. Likewise, when we evaluated embryonic development, the formation rates of blastocyst and hatched blastocyst on days 7 and 8/9 of culture, respectively, were not statistically different among the groups. A previous study reported that the addition of 10, 100, and 1,000 μM TUDCA during IVM of cumulus free oocytes did not increase fertilization and blastocyst formation rate when compared to those in the control group (Mochizuki et al., 2018). The mechanism behind the apparent decoupling of the effect of TUDCA on fertilization and blastocyst formation rates, which was observed despite the detection of beneficial markers (ER and oxidative stress mitigation), is unclear. The release of oscillatory calcium waves by spermatozoa phospholipase during fertilization (Ajduk et al., 2008; Ducibella and Fissore, 2008) only partially relies on a non-stressed ER. The ability of ER to supply calcium may be retained in the early stages of ER stress, and an alternative calcium source theoretically explains the observed decoupling. Moreover, the absence of effect (at least statistically) on blastocyst production could be attributed to the absence of beneficial TUDCA effect at the end of maturation after 7–9 days of embryo culture without TUDCA. A possible way to assess this possibility is to conjugate a TUDCA treatment both on IVM and IVC.

Although TUDCA treatment had no effect on the gene expression reported in ER stress, both in the oocyte and in the embryo, we cannot rule out its possible effect in relieving ER stress. Yoon et al. (2014) demonstrated that embryo development *in vitro* depended on the orchestration between ROS and ER stress. Similarly, our results demonstrated that oocyte treatment with 200 μM TUDCA, a supposed ER stress inhibitor, reduced ROS production and positively regulated the transcripts related to the antioxidant system. Moreover, the *in vitro* conditions used in this study (i.e., low O_2_ tension) were not strong enough to exert the TUDCA effect. A functional proof (i.e., pregnancy rate) can elucidate whether TUDCA effectively improves *in vitro* embryo production. Unlike our work, Khatun et al. (2020) observed that the addition of 100 μM of TUDCA to the maturation medium significantly decreased the expression of ER stress-related genes in bovine COCs (*GRP78*/*BIP*, *PERK*, *ATF4*, *IER1*, *XBP1*, and *CHOP*). However, we cannot make a direct comparison of these findings with our results, since the subspecies, protocol, and concentration of TUDCA (100 μM) investigated in that work were different from the experimental model proposed in the present work. Moreover, while Khatun et al. (2020) investigated gene expression in the COCs, our work evaluated specifically the relative abundance of target transcripts from oocytes.

In summary, TUDCA concentration was demonstrated to be decisive for the increase/decrease of *in vitro*-matured oocyte competence in our experimental model that used *B. taurus indicus* oocytes. The use of 200 μM TUDCA during IVM is suggested to be beneficial for relieving ER stress in oocytes through antioxidant effects. In contrast, supplementation with 1,000 μM TUDCA during IVM was detrimental to the development of oocytes and activated ER stress and mitochondrial apoptosis pathways. This study is just the second to report the action of TUDCA during IVM in bovine oocytes (Khatun et al., 2020); however, further studies are needed to confirm its possible influence on bovine subspecies, oxygen tension during incubation, and effect on the pregnancy rate of embryos produced.

## Data Availability Statement

The raw data supporting the conclusions of this article will be made available by the authors, without undue reservation.

## Ethics Statement

Ethical review and approval was not required for the animal study because the use of bovine ovaries from a commercial abattoir in Brazil does not require a previous approval of an ethical committee.

## Author Contributions

EP contributed to the conception and design of the study, collected and analyzed data, and wrote the manuscript. CC performed most of the experiments and analyzed the data. FF and PS performed RNA extraction and reverse transcription for cDNA of samples subjected to gene expression analysis. LB contributed to data analysis. GM and FP-L provided technical support and critically revised the manuscript. MN contributed to the conceptualization and design of the entire study and supervised and contributed to critical revision and intellectual input to the manuscript. All authors have read and approved the final manuscript.

## Conflict of Interest

The authors declare that the research was conducted in the absence of any commercial or financial relationships that could be construed as a potential conflict of interest.

## Footnotes

1 https://imagej.nih.gov/ij/index.html
