# Supplementary material for: Treatment of in vitro-Matured Bovine Oocytes With Tauroursodeoxycholic Acid Modulates the Oxidative Stress Signaling Pathway
Source: Front Cell Dev Biol. 2021 Feb 19;9:623852. doi: 10.3389/fcell.2021.623852 (PMC7933469; doi:10.3389/fcell.2021.623852)
Supplement: Supplementary file 1 [file Presentation_1.pdf]

Supplementary Material

Table S1. **Gene symbol, functions and primers assay ID used for microfluidic expression analyses (Biomark HD System - Fluidigm).**

Gene Symbol	Function	Assay ID*
**PPIA**	Reference gene	PA5-16914
**GAPDH**	Reference gene	Bt03210912_g1
**ACTB**	Reference gene	Bt03224617_g1
**RPL30**	Reference gene	Bt03226330_g1
**B2M**	Reference gene	Bt03251628_m1
**HMBS**	Reference gene	Bt03234763_m1
**HPRT1**	Reference gene	Bt03225311_g1
**RLP15**	Reference gene	Bt03288449_g1
**MAPK1**	Oocyte maturation	Bt03216718_g1
**BMP15**	Oocyte maturation	Bt03286494_u1
**H1FOO**	Oocyte maturation	Bt03228652_g1
**HS2**	Oocyte maturation	Bt03212695_g1
**PTX3**	Oocyte maturation	Bt03249011_m1
**VCAN**	Oocyte maturation	Bt03217333_m1
**NFE2L2**	Oxidative stress and response to cellular stress	Bt03817661_m1
**KEAP1**	Oxidative stress and response to cellular stress	Bt03228713_m1
**CAT**	Oxidative stress and response to cellular stress	Bt03215423_g1
**SOD1**	Oxidative stress and response to cellular stress	Bt03215423_g1
**SOD2**	Oxidative stress and response to cellular stress	Bt03244551_m1
**GPX1**	Oxidative stress and response to cellular stress	Bt03259217_g1
**GPX4**	Oxidative stress and response to cellular stress	Bt03259611_m1
**PRDX1**	Oxidative stress and response to cellular stress	Bt03223684_m1
**PRDX3**	Oxidative stress and response to cellular stress	Bt03214402_m1
**ARO(CYP19A1)**	Oxidative stress and response to cellular stress	Bt03213774_m1
**GFPT2**	Oxidative stress and response to cellular stress	Bt03250351_m1
**GLRX2**	Oxidative stress and response to cellular stress	Bt03229700_m1
**FOX3**	Oxidative stress and response to cellular stress	Bt03649334_s1
**TXNRD1**	Oxidative stress and response to cellular stress	Bt03215471_m1
**VNN1**	Oxidative stress and response to cellular stress	Bt03220248_m1
**H1F1A**	Oxidative stress and response to cellular stress	Bt03259341_m1
**HMOX1**	Oxidative stress and response to cellular stress	Bt03218624_m1
**EIF2A**	Endoplasmic reticulum stress	Bt03274460_m1
**HSPA5**	Endoplasmic reticulum stress	Bt03244880_m1
**HSPD1**	Endoplasmic reticulum stress	Bt04301470_g1
**HSPA1A**	Endoplasmic reticulum stress	Bt03292670_g1
**ATF4**	Endoplasmic reticulum stress	Bt03221057_m1
**ATF6**	Endoplasmic reticulum stress	Bt03287802_s1
**XBP1**	Endoplasmic reticulum stress	Bt03227621_g1
**DDIT3**	Endoplasmic reticulum stress	Bt03251320_g1
**DNMT1**	DNA methylation	Bt03224737_m1
**DNMT3A**	DNA methylation	Bt01027164_m1
**DNMT3B**	DNA methylation	Bt03259810_m1

**HP1**	DNA methylation	Bt03246076_m1
**PAF1**	DNA methylation	Bt03239371_g1
**NANOG**	Related to pluripotency and cell differentiation	Bt03220541_m1
**POU5F1(OCT4)**	Related to pluripotency and cell differentiation	Bt03223846_g1
**HAND1**	Related to pluripotency and cell differentiation	Bt04318733_g1
**REST**	Related to pluripotency and cell differentiation	Bt03278318_s1
**IGFBP2**	Related to embryo development and cell proliferation	Bt01040719_m1
**IGFBP4**	Related to embryo development and cell proliferation	Bt03259500_m1
**SOX2**	Related to embryo development and cell proliferation	Bt03278318_s1
**IFITM3**	Related to embryo development and cell proliferation	Bt03292973_g1
**GSK3A**	Related to embryo development and cell proliferation	Bt03273698_g1
**KRT8**	Related to embryo development and cell proliferation	Bt03225178_g1
**LUM**	Related to embryo development and cell proliferation	Bt03211920_m1
**MTIF3**	Related to embryo development and cell proliferation	Bt03231844_m1
**TNF**	Related to embryo development and cell proliferation	Bt03259156_m1
**S100A10**	Related to embryo development and cell proliferation	Bt03215645_m1
**S100A14**	Related to embryo development and cell proliferation	Bt03230771_g1
**GATM**	Related to embryo development and cell proliferation	Bt03237896_m1
**BAX**	Related to apoptosis	Bt03211777_g1
**CASP9**	Related to apoptosis	Bt04282453_m1
**CASP3**	Related to apoptosis	Bt03250954_g1
**BID**	Related to apoptosis	Bt03241255_m1
**CD40**	Related to apoptosis	Bt03817804_g1
**1L-1b**	Related to apoptosis	Bt03212740_m1
**NFKB2**	Related to apoptosis	Bt03272789_g1
**SREBF1**	Related to metabolism	Bt03276370_m1
**SREBF2**	Related to metabolism	Bt04283467_m1
**ACACA**	Related to metabolism	Bt03213360_m1
**ACSL3**	Related to metabolism	Bt04282138_m1
**ELOVL3**	Related to metabolism	Bt00907566_m1
**ELOVL5**	Related to metabolism	Bt03235956_m1
**ELOVL6**	Related to metabolism	Bt00907566_m1
**FADS2**	Related to metabolism	Bt03256255_g1
**FASN**	Related to metabolism	Bt03210471_g1
**AQP3**	Related to metabolism	Bt03253663_m1
**PGK1**	Related to metabolism	Bt03225854_mH
**SLC2A3**	Related to metabolism	Bt03259513_g1
**SLC2A5**	Related to metabolism	Bt03258299_g1
**AKR1B1**	Related to metabolism	Bt03218049_g1
**G6PD**	Related to metabolism	Bt03649181_m1
**AGPAT9**	Related to metabolism	Bt04292093_m1
**PPARA**	Related to metabolism	Bt03220821_m1
**PLIN2**	Related to metabolism	Bt03212182_m1
**PLIN3**	Related to metabolism	Bt03230537_m1

* ThermoFischer Scientific
